# The future of traumatic brain injury research

**DOI:** 10.1186/1757-7241-22-S1-A7

**Published:** 2014-07-07

**Authors:** Mark Wilson, Parjam Zolfaghari, Collete Griffin, David Lockey, Christos Tolias, Vishi Verma

**Affiliations:** 1 London’s Air Ambulance and the Pan London Neurotrauma Group, London, UK

## Introduction

Traumatic Brain Injury (TBI) research is evolving. The quest for level-1 evidence through randomised prospective interventional trials, while useful to establish safety and efficacy, is changing to an era of big data observational studies. Comparative Effectiveness Research (CER) utilises the observation of differences between treatments and centres without strict inclusion/exclusion criteria or formal intervention [1]. In addition, the transfer of monitoring technologies into the pre-hospital field will enable research into earlier, more critical phases of brain injury. These two changes in TBI research direction should enable a better understanding of the spectrum of TBI diseases and foster a future of precision, personalised TBI management.

## Global epidemic of TBI

Trauma is the commonest cause of death in under 45s and TBI the commonest cause of this death [2]. Many survivors sustain considerable morbidity with lifelong effects for them, their family and society. In the US approximately 5.3 million live with disability following TBI. In Europe, this number is estimated to be 7.7 million [3] with 30-70% suffering on-going mental illness [4]. Despite this, TBI fails to attract research funding appreciated by more recognisable diseases such as cancer and heart disease.

## Problems with current TBI research

### TBI classification

TBI is traditionally classified as mild, moderate or severe based on initial Glasgow Coma Score (GCS) however, clinical experience tells us this is inaccurate. Patients with a GCS of 15 can die from an extradural and GCS 3 patients can be normal post-epileptic seizure. Approximately 25% of “mild” head injuries do not return to work and 84% have problems one year after injury, questioning how “mild” these injuries really are. Such classification is not appropriate for precision research. TBI is not one disease but a heterogenous collection (extradural/subdural, diffuse axonal etc) the outcome of which is determined by multiple factors such as injury location, physiology, extracranial injuries, and constitutional effects of the patient. Similarly, treatments are heterogeneous varying between centres and clinicians. Statistical adjustments (e.g. for extracranial injuries) are often inadequate and large observational studies are often retrospective. Whilst these may confirm common sense (e.g. treatment at a neurosciences centre improves outcome [5]; they also run the risk of generating self-fulfilling prophecy (e.g. the belief elderly patients do badly alters their care [6].

### Interventional trials

“Level 1” evidence studies can be contrived for ethical or logistical purposes. Surgical intervention studies usually require surgeon equipoise before randomisation [7,8]. Do such results apply across the entire disease spectrum if only those in equipoise were entered? Neuroprotection drug administration within minutes of injury is difficult; hence most studies have a window of opportunity (e.g. 8 hours). Such studies have had a negative outcome [9,10], however, neurons die within minutes of ischaemia and such logistical delays may create a type II error masking a real neuroprotective effect if given early.

Most interventional trials dichotomise outcomes but this can be clinically irrelevant and statistically inefficient. For example, a patient expected to be in vegetative state, owing to intervention, moves to a severely disabled state. Two novel approaches, sliding dichotomy and proportional odd models, overcome these inefficiencies. Sliding dichotomy requires patient stratification according to baseline risk and a point of dichotomy is identified [11]. For patients stratified in poor prognosis group, survival might be most relevant, whereas for those with a good prognosis any outcome worse than good recovery is undesirable. Robust prediction models are essential for such strategies [12]. Currently 2 models are available: CRASH and IMPACT while good at predicting death, fail to predict functional outcome. A large observational study of 3626 patients attempting to validate these risk prediction models, concluded that the IMPACT model was well calibrated for 6 months mortality but substantially under-predicted the risk of unfavorable outcome [12]. Large purpose built registries will refine such models.

Currently a number of fundamental neurotrauma principals (e.g. ICP monitoring) are being questioned [13]. An open mind is required to think again through these basic tenants.

### Timing of studies

Most acute TBI research occurs on intensive care, a controlled safe environment. However, the time of greatest secondary injury is likely to be in the pre-hospital environment with hypoxia, hypotension, and expanding haematoma causing more neurological damage. It is this environment where intervention will have the greatest impact.

## The future of TBI research

### Big data

A pragmatic approach to TBI research is required. A large European observational study of 5400 TBI patients is about to start (http://www.center-tbi.eu). Using CER (across three strata of Emergency Department, Ward and Intensive Care admissions) and by studying treatment and outcome variations, best practice should be identified. Further collaboration with TrackTBI (the US TBI data portal) should harmonise TBI common data element collection.

### Pre-hospital research

Many pre-hospital interventions (intubation, thoracotomy, blood administration) have been introduced in services like London’s Air Ambulance. If pre-hospital neuropathology assessment could be achieved, then roadside personalised neurotrauma treatment may be possible. This has started in stroke medicine with dispatch of CT enabled ambulances to potential stroke patients [14]. On scene imaging enables rapid thrombolysis. Similarly earlier neuroprotective interventions may be possible with on scene TBI diagnosis.

### Pan-london neurotrauma group

London is in a unique position for TBI data collection. The capital (daytime population = 12 million) is serviced by four major trauma centres, each receiving from one ambulance service (London Ambulance Service) and one Air Ambulance (London’s Air Ambulance). Additionally, all four receive severely injured patients from South East England via county air ambulances. This results in a population coverage of 16-18 million. The capital contains some of the top UK academic institutions that also have areas of specific interest within brain injury research. The pan-london neurotrauma group (http://www.londonneuro.com) will coordinate both clinical guideline development and academic collaboration. By creating an on-going prospective neurotrauma registry future interventional studies will be easier to instigate and run.

## Conclusion

TBI is a major global problem, but new ways of studying it, both by collecting large quantities of data and by studying the early evolution of neurological injury offer the most exciting opportunities yet to better understand brain injury and provide disease targeted management plans.
